# Thinking inside and outside the box: Comparing the efficacy of camera trap designs for surveying small mammals

**DOI:** 10.7717/peerj.21654

**Published:** 2026-09-23

**Authors:** Phoebe Williams, Xenia Green, Amelia Grass, Anthony Caravaggi

**Affiliations:** School of Applied Sciences, University of South Wales, University of South Wales, Pontypridd, United Kingdom

**Keywords:** Small mammals, Camera traps, Detection efficacy, Biodiversity monitoring, Mammal ecology, Wildlife conservation, Passive monitoring techniques, Cryptic species detection, Field methods in ecology, Ecological monitoring tools

## Abstract

**Background:**

Small mammals are vital to ecosystem health and serve as key indicators of environmental changes. Population trends in the UK for many species have remained largely undocumented, however, emphasizing the need for more effective monitoring tools. This study evaluates the efficacy of three camera trap designs for detecting small mammals: no-box camera traps (NCTs), open box camera traps (OCTs), and closed tube camera traps (TCTs).

**Methods:**

The study was conducted at Silent Valley Nature Reserve, South Wales, focusing on *Fagus sylvatica* woodland. The authors thank the reserve manager for providing verbal permission to access the site managed by the Gwent Wildlife Trust. Six baited survey stations, each equipped with one NCT, OCT, and TCT, were established in low-disturbance areas, placed approximately 5 m apart. Cameras recorded 15-second videos with a five minute trigger interval. Generalised additive models (GAMs) were used to assess temporal detection patterns, while hourly detection data and overlap metrics evaluated similarities between apparent activity patterns across trap types.

**Results:**

Mice (*i.e.*, wood *Apodemus sylvaticus* and yellow-necked *A. flavicollis* mice; *n* = 827) and bank voles (*Myodes glareolus*; *n* = 454) were the most commonly detected species in 1,874 detections of small mammals across 57 camera trap days. Both boxed designs yielded more detections of focal species and fewer false triggers than NCTs. TCTs recorded greater number of focal species detections relative to total detections (79%; compared to 58% for OCTs and 14.8% for NCTs) and the fewest unidentified species (16, compared to 51 and 49 respectively). Vole and shrew activity patterns differed between boxed and NCT designs. There was evidence of differences in detection among box designs, with shrews being more frequently detected by TCTs (*n* = 196), and voles more frequently detected by OCTs (*n* = 273).

**Discussion:**

Boxed traps aided species identification but were affected by camera focal issues, which could be mitigated with low-cost adaptations. Our study highlights the need to consider focal species ecology when designing camera trap studies and provides a basis for the development of more effective small mammal surveys, survey methods and conservation tools.

## Introduction

Small mammals play essential roles in ecosystem functioning (*e.g.*, Michel, Burel & Butet, 2006; Kaufman, Kaufman & Brillhart, 2010; McCleery et al., 2014) and can be important indicators of change (Macdonald & Tattersall, 2001; Rowe & Terry, 2014). Multiple syntheses show a persistent taxonomic bias in biodiversity data and conservation research, however, with a heavy skew toward birds, plants and charismatic large vertebrates. Hence many small-bodied or less-charismatic groups are systematically under-represented (Boakes et al., 2010; Amano, Lamming & Sutherland, 2016; Di Marco et al., 2017; Troudet et al., 2017; Christie et al., 2020). This includes small mammals (*i.e.,* non-volant terrestrial mammals with an adult body mass of less than around 5 kg and typically comprise rodents, insectivores, and lagomorphs; Brown & Nicoletto, 1991; Wilson & Reeder, 2005; Merritt, 2010; Stoddart, 2012; Lim & Pacheco, 2016) as their diminutive size and often cryptic behaviour (Barros et al., 2024), make them challenging to study (Littlewood et al., 2021).

The UK is home to fifteen species of terrestrial small mammals. These include the bank vole *Myodes glareolus*, the field vole *Microtus agrestis*, common *Sorex araneus*, pygmy *Sorex minutus*, and water *Neomys fodiens* shrews, and wood *Apodemus sylvaticus,* and yellow-necked *Apodemus flavicollis* mice. A recent study suggested that small mammal populations are declining in the UK (Coomber et al., 2021), however many species are considered data-deficient (Mathews et al., 2018). For example, population trends for *S. araneus* and *S. minutus* are unknown (Battersby, 2005; Mathews et al., 2018), despite the species being important bioindicators (Sánchez-Chardi & López-Fuster, 2009) or vulnerable to displacement by invasive species (Browett et al., 2023). Effective survey methods are therefore required to fill important knowledge gaps (Marsh & Trenham, 2008).

Monitoring small mammals typically involves live trapping. This method often delivers high-quality data (McCain et al., 2018; Toms, Siriwardena & Greenwood, 1999) at the risk of inducing stress (Sikes et al., 2016), mortality (Do, Shonfield & McAdam, 2013), and potentially promoting behavioural change (Johnstone, Garvey & Hickling, 2023). As such, non-invasive methods such as footprint tunnels (Ellenbroek, 1980), hair tubes (Baker et al., 2003), and camera traps (Steenweg et al., 2016; Wearn & Glover-Kapfer, 2019; Fennell, Beirne & Burton, 2022; but see Caravaggi et al., 2017) are increasingly used.

The application of camera traps to the detection of small mammals has increased over time (*e.g.*, Meek, Vernes & Falzon, 2013; Burns et al., 2018; Dundas et al., 2019) yet the efficacy of camera traps to accurately detect small mammals has not been sufficiently assessed (Thomas et al., 2020) and several challenges remain. For example, the size and speed of small mammals make them difficult to detect and identify (Glen et al., 2013). Smaller mammals may also be less likely to trigger camera trap sensors (Klemens, Tripepi & McFoy, 2021) and identification can be challenging, particularly with morphologically similar species (Meek, Vernes & Falzon, 2013; McKibben & Frey, 2021). In camera trap studies of small mammals, species are typically distinguished using a combination of gross morphological traits including relative body size, tail-to-body length ratios, pelage coloration, and general body shape, often inferred from posture and movement within the field of view. However, these traits can be difficult to assess reliably in conventional camera trap setups where animals are partially obscured, captured at oblique angles, or recorded at distance against complex backgrounds. Advances in the optimisation of camera traps for the study of small mammals (*e.g.*, Glen et al., 2013; Hobbs & Brehme, 2017; Gracanin, Gracanin & Mikac, 2018; Fink & Jachowski, 2025) are yielding new opportunities for research to help close knowledge gaps. These advancements include the development of boxed camera traps that aim to mitigate these limitations by intervening in each stage of this detection process (*e.g.*, Littlewood et al., 2021; Soininen et al., 2015; Dueser, Porter & Moncrief, 2025). By providing a confined and baited micro-environment, boxed systems may increase the likelihood that animals enter the sensor detection zone at close range, thereby overcoming distance-related detection failure and improving trigger reliability. The presence of bait within a restricted and partially enclosed space further increases residence time, reducing the likelihood that individuals pass rapidly through the field of view before capture. In addition, confinement within the box or tunnel promotes stationary or slow-moving behaviour and positions individuals centrally within the frame, improving image quality and the likelihood of diagnostic feature capture. Boxed cameras also offer distinct advantages in that they can be adapted to suit different camera models, and they do not require technical manipulation that may void warranties or make cameras inoperable. In addition, their enclosed structure may provide a sheltered environment for small mammals (*e.g.*, Littlewood et al., 2021; Fink & Jachowski, 2025).

Here we compared the efficacy of three different camera trap designs—a camera trap without a box (‘no box’; NCT), an open box camera trap (OCT; Littlewood et al., 2021), and a closed camera trap box with tube entry (TCT; adapted from Mos & Hofmeester, 2020)—for the detection and study of small mammals. We hypothesized that the TCT would yield the greatest number of small mammal detections as their constrained field-of-view and funnel-like structure increase the probability that individuals enter the detection zone and are successfully recorded. We also explored the potential applicability of the camera trap data gathered to analyse small mammal activity patterns.

## Materials and Methods

**Figure 1:**
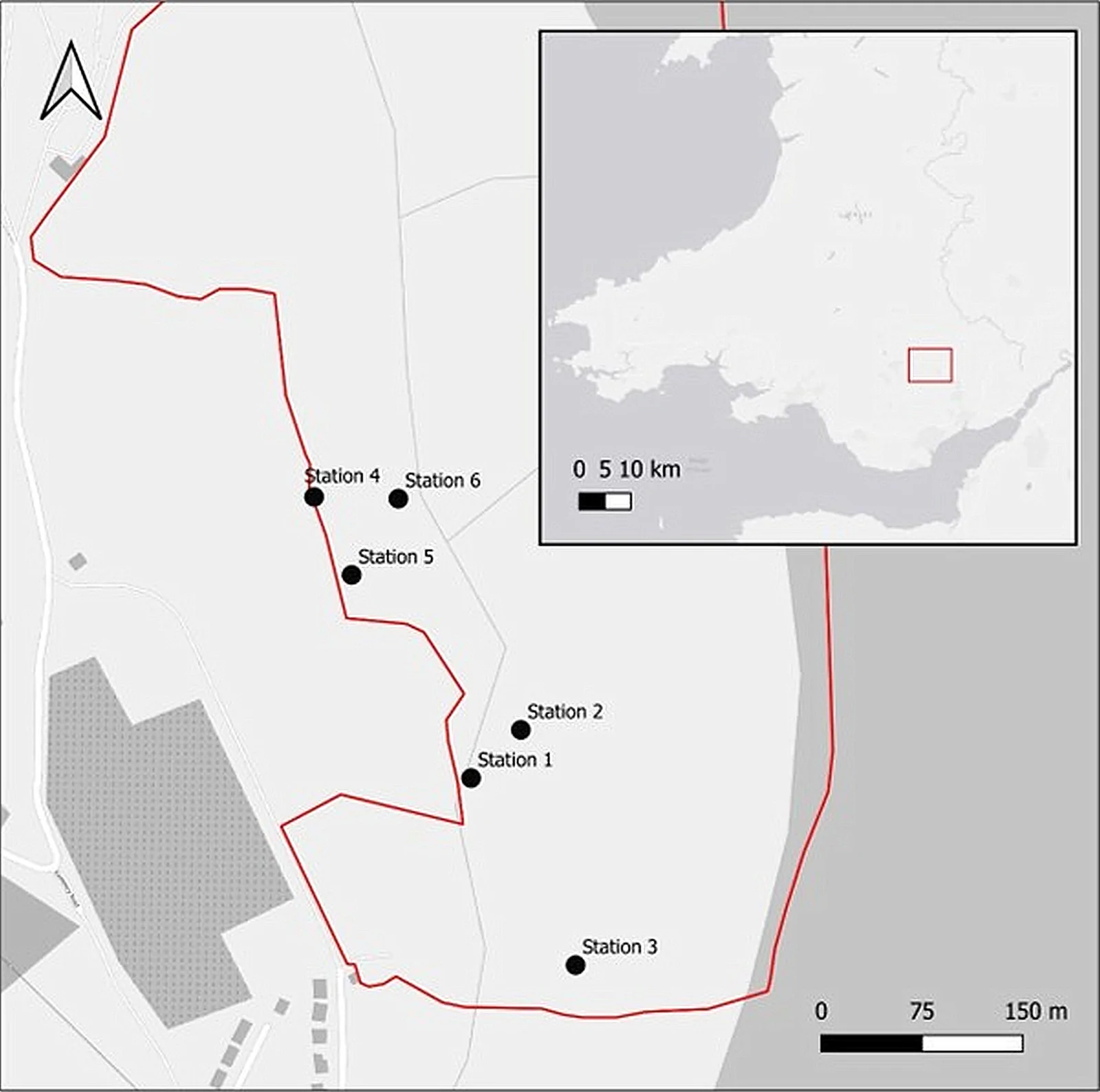
Locations of survey stations in Silent Valley Nature Reserve, Ebbw Vale, South-east Wales. Each station comprised three traps–one with no box, one open box, and one tube box.

The study was undertaken at Silent Valley Nature Reserve, South Wales (51°44′57.48″ N, 003°10′42.59″W; Fig. 1). The reserve comprises approximately 50 ha that includes beech *Fagus sylvatica* woodland, upland heath, and unimproved rough grassland. Part of the reserve, Cwm Merddog Woodlands, is designated a Site of Special Scientific Interest (SSSI) (NRW, 1994). Through the reserve runs Nant Merddog, a tributary of the river Afon Ebbw Fawr. Six survey locations were identified based on focal species ecologies and field signs, and in areas away from high footfall. All locations were in *F. sylvatica* woodland. Each survey station was comprised of three camera traps, one each of NCT, OCT, and TCT. Traps within each survey station were spaced approximately 5 m apart and positioned to increase the chance of encountering small mammals (*e.g.*, by placing traps alongside logs, runways, or possible sites for shelter; Cunningham et al., 2005). Camera traps were a mixture of six Bushnell 119440 Nature View HD Max (NCTs), and twelve Victure HC200 Hunting Cameras (OCTs and TCTs; 73° perspective, 0.5 s trigger speed), all of which used infra-red (850 nm) for illumination. The variation in camera traps used was due to the relative availability of equipment, and time restrictions to complete the study. Cameras were tested *via* paired trials to ensure comparable detection and image quality prior to deployment. Any potential model effects were accounted for by including camera type as a covariate in subsequent analyses (Rovero et al., 2013).

**Figure 2:**
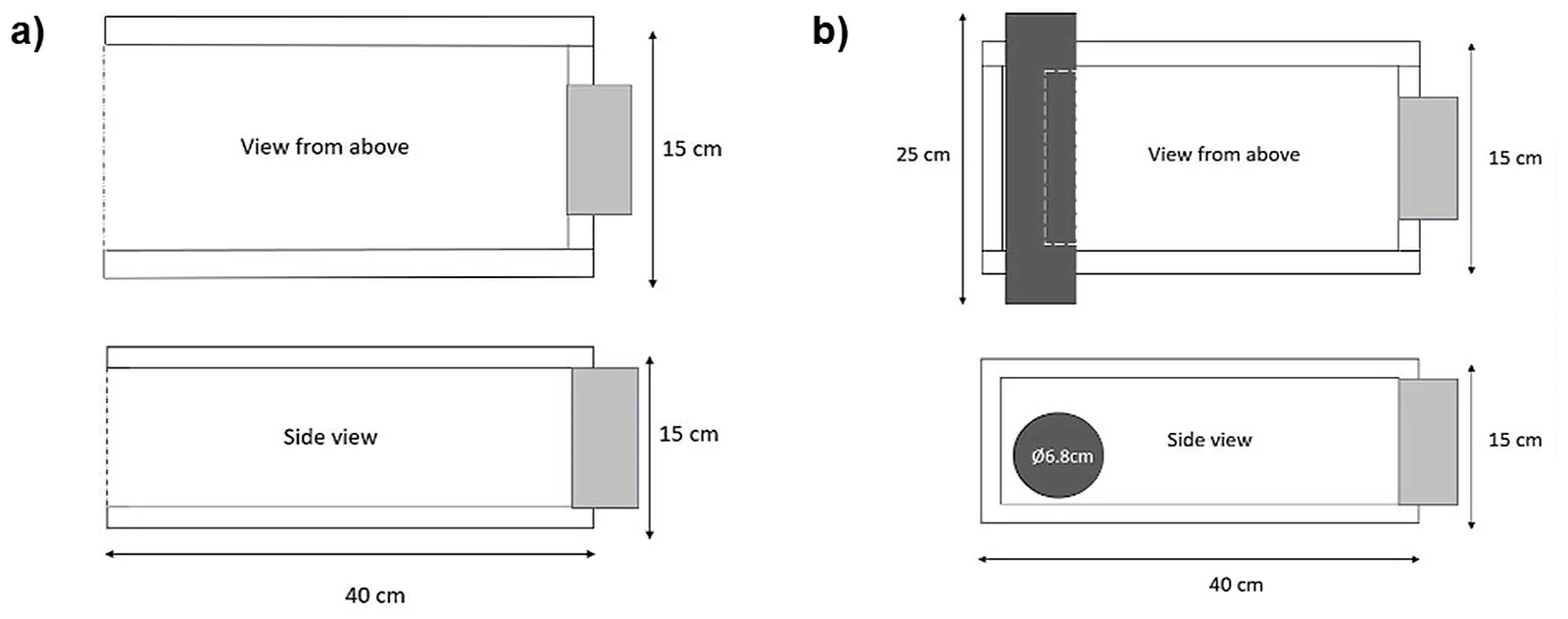
Design specifications for (A) ‘open box’ and (B) ‘tube box’ camera trap designs. The tube box was an adaptation of the *mostela* by Mos & Hofmeester (2020). Light grey rectangle = camera trap. Dark grey rectangle/circle = UPVC pipe.

Camera traps were set to record 15-second videos at each trigger, with a delay of five minutes between triggers (Littlewood et al., 2021). Videos were recorded to provide continuous temporal context (*i.e.,* movement trajectories, behaviour) for further study, along with clearer identifications of cryptic species. The camera trap sensitivity was set to automatic. NCTs were mounted so that the sensor and lens were 30 cm from the ground (Glen et al., 2013) and at an angle of approximately −15 degrees from horizontal. OCTs were housed in a wooden box that was open on one end. TCTs were housed in a closed box with a 25 cm-long uPVC tube (diameter = 6.8 cm) serving as a double-ended entrance/exit, adapted from Mos & Hofmeester (2020) (Fig. 2). All boxes measured 40 cm × 15 cm × 15 cm and were made with oriented strand board, with camera traps attached to the end of each tunnel using wire. The camera sensor was positioned just above the floor of the tunnel, oriented horizontally, with the distal half of the tunnel in focus. All camera trap deployments were baited to standardise detection probability across designs and to increase encounter rates of small mammals within the field of view. Baiting is widely used in camera trap studies to increase detection rates and reduce zero inflation, particularly for small or cryptic species where unassisted encounter rates may be low (Meek et al., 2014; Rovero et al., 2013; O’Connell, Nichols & Karanth, 2011). While enclosed or semi-enclosed structures such as boxed or tunnel traps may be used by small mammals as natural cover or movement corridors in the absence of bait, unbaited deployments typically yield lower detection probabilities and reduced analytical efficiency for comparative inference (O’Connell, Nichols & Karanth, 2011; Burton et al., 2015). In this study, baiting therefore functioned primarily as a means of standardising detectability across camera trap types rather than eliciting fundamentally different behavioural responses among treatments. Bait was placed into the distal third of the boxes. At NCT deployments, bait was placed on the ground surface approximately 30 cm from the camera lens and was fully exposed to ambient environmental conditions. Unlike boxed and tunnel deployments, bait at NCTs was not physically protected from removal by non-target species or environmental degradation. However, no systematic evidence of complete bait depletion by large mammals was observed. Bait was placed into the distal third of the boxes. This composed approximately 20 ml of a standard garden bird seed mix which included dried mealworms (Littlewood et al., 2021). A pilot study was used to identify appropriate bait placement and consider whether image quality was sufficient to detect and discriminate between small mammal species. Survey stations were active between the 11th of July and the 6th of September 2021. Some vegetation clearance was attempted when cameras were deployed but this was limited to mitigate impacts on small mammal behaviour (Apps & McNutt, 2018).

Both *A. sylvaticus* and *A. flavicollis*, were known to be present hence were grouped as *mice* due to difficulties with identification. Similarly, all voles and shrews were grouped accordingly for statistical analyses.

All statistical analyses were undertaken in R v. 4.3.3 (R Core Team, 2024). generalised additive models (GAMs, *via* the *mgcv* package; Wood, 2025) were used as a diagnostic tool to assess whether detection rates were temporally constant across the sampling period. The data were hierarchically structured, with three camera traps deployed within each of six survey stations, resulting in spatial clustering of sampling locations at approximately five m intervals. To account for this non-independence, station identity was included as a random effect in all models, thereby controlling for shared environmental conditions and spatial clustering within stations. In addition, an observation-level random effect was included to account for residual overdispersion and repeated detections. While a fully nested structure (camera within station) was considered, this was not implemented due to the limited number of stations (*n* = 6), which constrained model identifiability and risked overparameterisation. Consequently, inference is made at the level of treatment (camera trap design), with cameras treated as repeated sampling units within stations rather than fully independent spatial replicates. Count was fitted as the dependent variable and week was fitted as the smoothed explanatory variable. We used a thin plate smooth with the default number of knots. We compared three candidate models representing (i) no temporal structure, (ii) a shared temporal trend across treatments, and (iii) treatment-specific temporal trends. Model comparison (AIC) supported the shared-trend model, with only weak support for treatment-specific temporal variation.

Activity patterns were explored according to the number of detections of each species group per hour, beginning at HH:00:00 and ending at HH:59:59. Overlap metrics were used to examine apparent differences between camera trap types for each species, using the *overlap* package (Meredith & Ridout, 2014). Data were resampled to 1,000 iterations to produce 95% confidence intervals (CIs). Low overlap was defined as Δ ≤ 0.50, moderate overlap as 0.50 < Δ ≤ 0.75, and high overlap as Δ > 0.75 (Monterroso, Alves & Ferreras, 2014).

### Ethical statement

This study underwent a light-touch ethics review at the University of South Wales that determined that a full formal application was not required due to the nature of the research. As such, no application or permit number was issued. The study complied with institutional policies for low-risk wildlife research and adhered to relevant national and international guidelines, including the UK Animals (Scientific Procedures) Act 1986 (as amended 2012) and the EU Directive 2010/63/EU on the protection of animals used for scientific purposes. Access to the field site was approved by the Silent Valley Nature Reserve manager; no documentation was provided.

We followed the 3Rs principles (Replacement, Reduction, Refinement) by employing non-invasive infra-red, no-flash camera traps to observe small mammals without capture, handling, or physical interference. The study design was refined to minimise potential disturbance, and footage consistently showed animals engaging in natural behaviours such as foraging and feeding, with no evidence of stress or avoidance.

In line with taxon-specific ethical standards, we followed the guidelines of Sikes et al. (2016) for the use of wild mammals in research and education. We also considered welfare-focused recommendations (Nogues, Arends & von Keyserlingk, 2025) regarding the deployment and potential impacts of camera traps in ecological research. Footage showed no signs of distress or avoidance in detected animals that frequently engaged in natural behaviours, including feeding at bait stations. This ethics statement was prepared in accordance with the ARRIVE 2.0 guidelines for transparent reporting of animal research Du Sert et al. (2020a), Du Sert et al. (2020b).

## Results

### Detections

A total of 4,025 triggers were recorded across 57 camera trap days (*i.e.,* 24-hour periods), 2,392 (60%) of which were detections of animals; the rest were false triggers (*i.e.,* an image with no immediate evidence of being triggered by an animal). Focal species accounted for 1,874 detections of which: mice comprised 44.1%; bank voles were 24.2%; pygmy shrew made up 9.3%; common shrew comprised 8.1%; field vole made up 3.3%; and water shrew comprised <1%. Non-target species—including 12 bird species, seven species of mammal, and one reptile—accounted for 27.6% of all detections (Table 1; Table S1.1).

**Table 1:** Detections and false triggers from three different camera trap designs. Detections of ‘other animals’ (i.e., birds, mammals, and reptiles) are given in the Supplementary Materials.

	**Camera trap build**			
	**No box**	**Open box**	**Tube box**	**Total**
Total triggers	1,544	1,498	983	4,025
False triggers	851	582	200	1,633
Small mammal detections	229	868	777	1,874
Detections of other animals	464	47	6	517

A total of 1,498 detections were recorded by OCTs, including 916 detections of animals and 582 false triggers. Focal species accounted for 868 of all animal detections (Table 1). Species recorded with this method included *mice* (47.9% of all focal species detections), bank vole (30.5%), pygmy shrew (7.7%), common shrew (6.2%), *voles* (0.8%), *shrews* (0.8%), and field voles (0.1%) (Table 2). A total of 51 small mammal detections could not be identified to genus or species level due to blurred or incomplete images. OCTs also detected a single brown rat *Rattus rattus* (Table S1.1).

**Table 2:** Total number of detections of small mammal focal species from three camera trap designs.

	**Species**		**Camera trap build**			
**Group**	**Common name**	**Scientific name**	**No box**	**Open box**	**Tube box**	**Total**
Mice	**Total**		98	416	313	827
Voles	Bank vole	*Clethrionomys glareolus*	5	265	184	454
	Field vole	*Microtus agrestis*	0	1	60	61
	Vole sp.	-	7	7	8	22
	**Total**		12	273	252	537
Shrews	Common shrew	*Sorex araneus*	13	54	85	152
	Pygmy shrew	*Sorex pygmaeus*	3	67	105	175
	Shrew sp.	-	52	7	6	65
	Water shrew	*Neomys fodiens*	2	0	0	2
	**Total**		70	128	196	394
Unidentified	**Total**		49	51	16	116
	**Grand total**		**229**	**868**	**777**	**1,874**

A total of 983 detections were recorded by TCTs, including 783 detections of animals and 200 false triggers. Focal species accounted for 777 of all animal detections (Table 1). Species recorded during this method included *mice* (40.2% of all animal detections), bank vole (23.6%), pygmy shrew (13.5%), common shrew (10.9%), field voles (7.7%), *voles* (1.02%), and *shrews* (*n* = 6, 0.8%). A total of 16 (0.2%) small mammal detections could not be identified to genus or species level (Table 2). TCTs recorded the fewest detections of non-focal species but did record the only instance of a weasel (*Mustela nivalis*) (Table S1.1).

A total of 1,544 detections were recorded by NCTs, including 693 animal detections and 851 false triggers. Focal species accounted for 229 of all triggers (Table 1). *Mice* (*n* = 98, 42.8% of all animal detections) were the most commonly detected small mammal species, followed by *shrews* (*n* = 52, 22.8%), common shrew (*n* = 13, 5.7%), *voles* (*n* = 7, 3.1%), bank vole (*n* = 5, 2.2%), pygmy shrew (*n* = 3, 1.3%) of detections, and water shrew (*n* = 2, 0.9%). A total of 49 (21.4%) small mammals were unidentifiable (Table 2).

**Figure 3:**
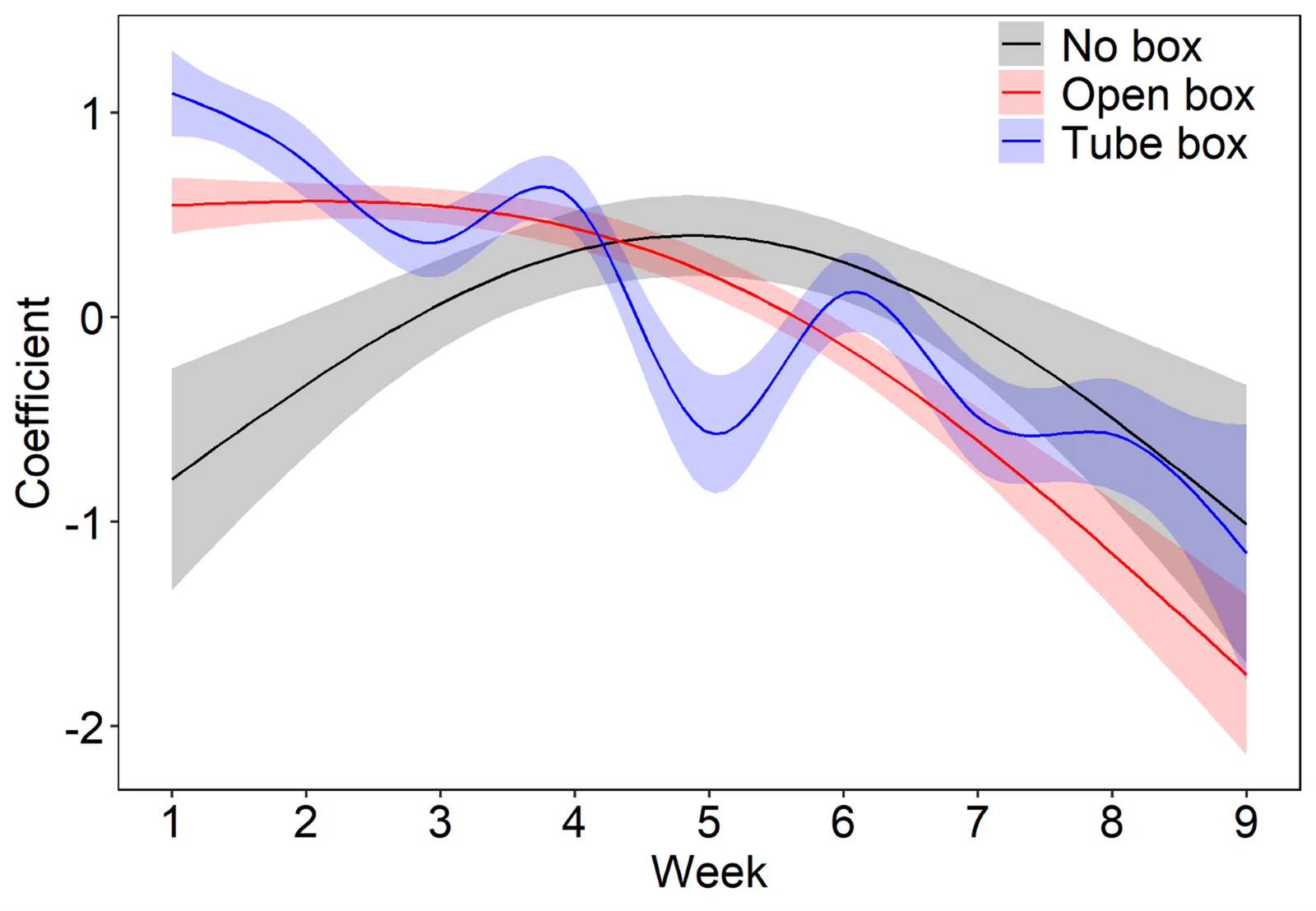
Generalised Additive Model (GAM) fit of camera trap detections of small mammals per week, for each of three different camera trap designs.

All GAMs showed significant temporal variation in detection frequencies for each trap design (NCT *P* = 0.004; OCT *P* < 0.0001; TCT *P* < 0.0001). Patterns from NCTs were visibly different to the boxed designs. NCT detections described a unimodal smoothed curve whereas OCTs and TCTs described a decline in detection probability over time. TCT data showed the greatest variance per week (Fig. 3).

### Activity patterns

**Figure 4:**
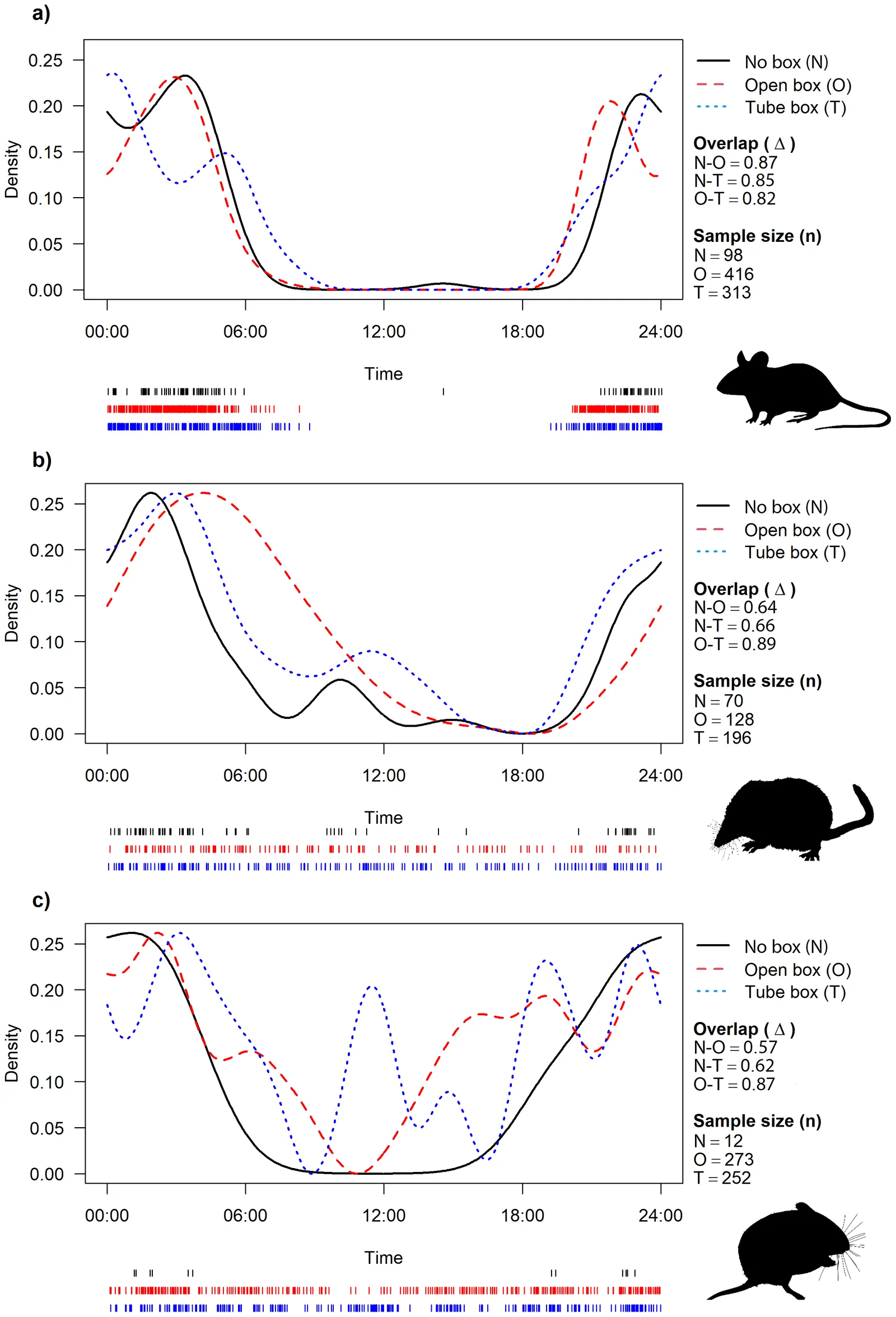
Activity patterns of (A) mice, (B) = shrews, and (C) = voles using three camera trap designs. Overlap metrics, sample sizes, and rugs indicating individual detections are given for each.

*Mice* were observed to be nocturnal, across all camera trap data, with 93% (88% TCTs –99% NCTs) of all detections occurring between 21:00 and 07:00 h. Activity patterns from NCTs and OCTs overlapped by 87.4% (95% Confidence Intervals (CI) [87.2–87.6%]), NCTs and TCTs overlapped by 84.9% (95% CI [84.7–85.1%]), and OCTs and TCTs had an overlap of 82.4% (95% CI [82.2–82.5%]) (Fig. 4A).

*Shrews* were largely nocturnal, with 59% of all activity between the hours of 21:00 and 07:00, however there were differences between camera trap types. NCTs showed a strongly nocturnal signature with 86% of detections occurring between 21:00–07:00. TCTs and OCTs were more likely to detect shrew in daylight hours, with 47% and 46% of detections respectively occurring between 07:00–21:00. Overlap between activity patterns was 64.0% (95% CI [63.6–64.4%]) for NCTs and OCTs, 66.4% (95% CI [66.0–66.7%]) for NCTs and TCTs, and 88.5% (95% CI [88.3–88.7%]) for OCTs and TCTs (Fig. 4B).

*Voles* were cathemeral, with irregular activity peaks that differed between camera trap type. Activity patterns from NCTs and OCTs had a moderate overlap of 57.2% (95% CI [56.6–57.7%]). NCTs and TCTs overlapped by 61.8% (95% CI [61.3–62.4%]). There was high degree of overlap in activity between OCTs and TCTs of 87.4% (95% CI [87.3–87.6%]) (Fig. 4C).

## Discussion

Camera trap studies of small mammals promise to reveal important insights into species ecologies. In this study we demonstrate that going beyond simple camera adjustments and instead providing species-appropriate structures can improve detection performance (also see Caravaggi et al., 2020; Mos & Hofmeester, 2020; Fink & Jachowski, 2025; Dueser, Porter & Moncrief, 2025). Both boxed camera types were recorded more detections than NCTs, and OCTs were generally detected more small mammals (*n* = 868), particularly *mice* (*n* = 416) and *voles* (*n* = 265). TCTs performed similarly (*n* = 777) and detected more *shrews* (*n* = 196) while also recording the highest number of field vole detections (*n* = 60).

Activity overlap analyses indicated that for some taxa, activity patterns were highly consistent across camera trap designs, suggesting that inference about activity patterns may be relatively robust to camera trap design. For example, mice showed high overlap across all trap types (82.4–87.4%), with all methods consistently indicating strongly nocturnal behaviour. This divergence occurred alongside substantial variation in detections among trap types (NCT = 12, OCT = 273, TCT = 252), indicating that unboxed cameras yielded considerably fewer observations of vole activity compared with boxed designs. This disparity suggests differences in detectability and use of camera trap structures rather than true differences in underlying behaviour and highlights the importance of considering sampling design when interpreting activity estimates. Shrews showed similar divergence among trap types, with lower overlap between NCTs and boxed cameras (64.0–66.4%) compared with OCT–TCT agreement (88.5%). However, NCTs suggested a more strongly nocturnal pattern (86% of detections between 21:00 and 07:00) than boxed designs, where a higher proportion of detections occurred during daylight hours (46–47%). This indicates that trap design may influence estimates of activity distribution for this group, with enclosure-based designs producing more consistent temporal profiles and unboxed deployments potentially biasing estimates of diel activity due to differences in detection probability across light conditions and microhabitats.

Previous studies have shown that mice are largely nocturnal (*e.g.*, Wolton, 1983; Caravaggi et al., 2018), a pattern consistently observed in our data. Vole activity differed between NCT (nocturnal) and boxed (cathemeral, consistent with previous research; (Eppley & Donati, 2019; Halle, 2006) camera trap types. Only 12 vole detections were recorded from NCTs, however, which is insufficient for the reasonable estimation of activity (Rowcliffe et al., 2014), but see (Frey et al., 2014). Similar inconsistencies between boxed and NCT designs were observed in the shrew data. Boxed camera traps showed that shrews exhibited greater levels of activity during the night and early hours of the morning. In contrast, data from NCTs suggested that shrews are strongly nocturnal. Shrews often display ultradian rhythms (*i.e.,* where periodicity can range from minutes to hours; (Rychlik, 2005) with seasonal (Pernetta, 1977) and temperature-induced variance (Churchfield, 1982). NCTs do not provide shelter like boxed camera traps and movement of small mammals across open space may be less likely than in sheltered areas or along runways (Dickman & Doncaster, 1987). However, NCTs detected the highest number of non-focal species. Disturbance by other animals may have impacted small mammal behaviour at NCTs, reducing the likelihood of detection (McCleery et al., 2014).

NCTs recorded the greatest proportion of false triggers, with 55% of detections not recording an animal. False triggers were likely caused by moving vegetation, or movement of an animal on the periphery of or rapidly transiting through the zone of detection. Such instances may lead to underestimates of animal presence (Jumeau, Petrod & Handrich, 2017) and consequently may impact the accuracy of population metrics (Foster & Harmsen, 2012) or the appropriate spatial allocation of conservation efforts (Gu & Swihart, 2004). False triggers also increase battery drain, reduce SD card capacity (Newey et al., 2015), and result in increased effort in terms of data processing. Boxed cameras were less prone to disturbances resulting in fewer false triggers (38% for OCTs and 20% for TCTs). With vegetation movement or light fluctuations most probably responsible for false triggers in OCTs (Wei et al., 2020). The enclosed nature of TCTs likely reduced exposure to these sources of interference, with false triggers more likely due to individuals passing rapidly through the tube without being captured or remaining just outside of the camera’s field of view. Because bait may attract small mammals to camera-trap stations (*e.g.*, Mills, Godley & Hodgson, 2016; Littlewood et al., 2021), maintaining bait availability could potentially increase detections; however, the effect of re-baiting frequency remains uncertain (*e.g.*, Sharpe et al., 2026). More frequent re-baiting would increase the number of human visits, however, disturbing surrounding vegetation and creating obvious pathways to camera trap locations which may result in camera theft (Meek, Ballard & Falzon, 2016) and data loss (Caravaggi et al., 2017).

Although the no-box camera trap was the least effective method, it was the only survey method that recorded water shrew. It is unclear why the species was not detected on boxed cameras as the species is of comparable size (*i.e.,* up 10 cm in length and 20 g in weight) to others detected in this study. While environmental covariates were not considered in this study, temporal variation in detection rates (as identified in the GAMs) provides evidence that detection probability and/or local activity was not constant over the survey period. This is relevant because differences in camera trap design may interact with temporal shifts in behaviour (*e.g.*, changes in foraging activity or resource use), potentially influencing apparent relative performance among trap types. Indeed, GAM outputs indicate non-linear variation in detections through time, suggesting that sampling was not temporally uniform and that peak detection periods may differ between camera designs. This implies that comparisons of total detections between trap types should be interpreted cautiously, as differences may partially reflect temporal variation in detectability rather than only structural differences among trap designs. Other factors that should also be considered include phenology (*e.g.*, breeding period, Wilson, Montgomery & Elwood, 1993; Churchfield, Hollier & Brown, 1995), predation risk (Ylonen & Ronkainen, 1994; Korpimaki et al., 2002), and climate (Santoro et al., 2017).

The effective use of camera traps for small mammal surveys hinges on the capacity of the observer to confidently identify individuals to species level and, for population metrics, distinguish individuals (Thomas et al., 2020). Traits such as relative tail and body length are often used but are difficult to quantify within the wide field of view of a traditional camera trap setup and with poor contrast between the animal and the background (DeBondi et al., 2010). The design of TCTs—that returned the fewest unidentifiable detections (*n* = 16)—ensured that individuals entered the box in profile, providing the opportunity to gauge relative tail and body length. The black tubes also provided contrast between individuals and the background in most instances, though some difficulties remained. White tubes with scale bars (Mos & Hofmeester, 2020) are therefore suggested for future studies.

Previous studies suggest that small-scale habitat features*,* and feature quality are important considerations (Williams, Marsh & Winter, 2002; Kolowski & Forrester, 2017; McCarthy et al., 2021). This study deliberately selected stations in good-quality habitat, aligned with notable field signs of the target species, as is common for small mammal surveys (Flowerdew et al., 2004). Traps were also baited to keep individuals in front of the camera long enough to support species identification (Littlewood et al., 2021). This increased detection probabilities across camera trap stations but can also result in incorrect estimates of detection and occupancy (Fidino et al., 2020). Consequently, temporal variation captured in the GAM analyses should be interpreted as a combination of true variation in activity and variation in bait-mediated detection probability. However, as baiting was applied consistently across all camera trap types, any systematic bias introduced by bait is expected to be comparable among treatments, allowing robust relative comparisons of temporal patterns and trap performance.

Further, identification may have been inhibited using black and white imagery, obscuring diagnostic features such as the distinctive yellow band on the neck of *A. flavicollis,* absent in *A. sylvaticus.* White flashes are not recommended for camera trap studies as they can impact behaviour in detected individuals (Caravaggi et al., 2017). Moreover, both boxed designs highlighted issues regarding the proximity of focal animals to the camera, where only parts of individuals were visible and/or clarity (*i.e.,* focal length) was compromised. Low-cost adaptations such as the use of close-focus filters (Klemens, Tripepi & McFoy, 2021) or additional lenses (*e.g.*, as accompany the Wilsus Juxta Close-Focus model) may mitigate this issue. Complementary methods, like further adaptation of TCTs to collect hair samples (McKelvey & Pearson, 2001) could aid in identification and the application of capture-mark-recapture models (Kery et al., 2010; Croose et al., 2019), though this would incur further cost and analytical effort. Finally, novel camera trapping designs could also be used in conjunction with the traditional boxed and unboxed traps to enhance small mammal monitoring (Fink & Jachowski, 2025; Dueser, Porter & Moncrief, 2025).

Finally, two camera models were used across deployments. Although all camera trap designs were deployed in a fully crossed, within-station arrangement, camera model effects could not be statistically separated from residual variation in detection probability given the limited replication available. Exploratory hierarchical models indicated substantial uncertainty in camera-specific parameters and poor identifiability, suggesting that any model-level differences are minor relative to overall detection variability in this dataset. However, this limitation does not compromise the primary objective of the study, which is a paired comparison of detection performance among camera trap designs under identical spatial and temporal conditions. The within-station design ensures that any unmeasured camera-specific heterogeneity is shared across treatments, thereby preserving the validity of relative comparisons among camera types. Future studies aiming to isolate camera model effects should incorporate dedicated calibration trials or increased replication to allow independent estimation of device-level performance.

## Conclusion

Small mammals have crucial roles within ecosystems. There are substantial knowledge gaps, however, and there is a need to identify survey techniques that are effective and can be used by volunteers, ecological professionals, and researchers alike. This study demonstrates that boxed camera traps, which offer protection from inclement weather and, potentially, predators, represent a potential improvement on NCTs. TCTs were particularly effective with fewer unidentified individuals and fewer false triggers. There was some evidence of differential box preference between species, with shrews being more frequently detected in TCTs, and voles more frequently detected by OCTs. Our findings will prove valuable for those involved in small mammal conservation, management, and research and support the development of more effective surveys and the collection of crucial data.

## Supplemental Information

10.7717/peerj.21654/supp-1 Supplemental Information 1 Non-target species recorded by three different camera trap designs at Silent Valley Nature Reserve

## Additional Information and Declarations

### Field Study Permissions

The following information was supplied relating to field study approvals (i.e., approving body and any reference numbers): Tom Eyres, reserves officer of the Gwent Wildlife Trust, who had authority over Silent Valley Nature Reserve, approved researcher access to their site for this work.

### Data Availability

The following information was supplied regarding data availability: The code and data are available at Zenodo: Anthony Caravaggi. (2025). arcaravaggi/2024_smammal_cts: v1.0.0 (Versions 2024_smammal_cts_1) (Computer software). Zenodo. https://doi.org/10.5281/zenodo.16355322
