# Supplementary material for: Thinking inside and outside the box: Comparing the efficacy of camera trap designs for surveying small mammals
Source: PeerJ. 2026 Sep 23;14:e21654. doi: 10.7717/peerj.21654 (PMC13615732; doi:10.7717/peerj.21654)
Supplement: Supplementary file 1 [file peerj-14-21654-s001.docx]

**Supplementary Materials**

**Table S1.1.** Non-target species recorded by three different camera trap designs at Silent Valley Nature Reserve.

|  | **Species** | |  | **Camera trap design** | | |  |
| --- | --- | --- | --- | --- | --- | --- | --- |
| **Group** | **Common name** | **Scientific name** |  | **No box** | **Open box** | **Tube box** | **Total** |
| **Bird** | Blackbird | *Turdus merula* |  | 9 | 0 | 0 | 9 |
|  | Bullfinch | *Pyrrhula pyrrhula* |  | 1 | 0 | 0 | 1 |
|  | Dunnock | *Prunella modularis* |  | 74 | 3 | 0 | 77 |
|  | Great Tit | *Parus major* |  | 2 | 0 | 0 | 2 |
|  | Green Woodpecker | *Picus viridis* |  | 1 | 0 | 0 | 1 |
|  | Grey Wagtail | *Motacilla cinerea* |  | 1 | 0 | 0 | 1 |
|  | Robin | *Erithacus rubecula* |  | 77 | 7 | 0 | 84 |
|  | Siskin | *Spinus spinus* |  | 1 | 0 | 0 | 1 |
|  | Song Thrush | *Turdus philomelos* |  | 54 | 5 | 0 | 59 |
|  | Thrush sp. | *Turdus* sp. |  | 2 | 0 | 0 | 2 |
|  | Unidentified | - |  | 44 | 10 | 0 | 54 |
|  | Wren | *Troglodytes troglodytes* |  | 30 | 19 | 2 | 51 |
|  | **Total** |  |  | **296** | **44** | **2** | **342** |
| **Mammal** | Brown Rat | *Rattus rattus* |  | 6 | 1 | 3 | 10 |
|  | Domestic Cat | *Felis catus* |  | 2 | 1 | 0 | 3 |
|  | Domestic Sheep | *Ovis aries* |  | 40 | 0 | 0 | 40 |
|  | Eurasian Hedgehog | *Erinaceus europaeus* |  | 1 | 0 | 0 | 1 |
|  | Grey Squirrel | *Scirius carolinensis* |  | 108 | 0 | 0 | 108 |
|  | Red Fox | *Vulpes Vulpes* |  | 11 | 0 | 0 | 11 |
|  | Weasel | *Mustela nivalis* |  | 0 | 0 | 1 | 1 |
|  | **Total** |  |  | **168** | **2** | **4** | **174** |
| **Reptile** | Common Lizard | *Zootoca vivpara* |  | 0 | 1 | 0 | 1 |
|  | **Grand total** |  |  | **464** | **47** | **6** | **517** |
